# Point-of-Care Urine Tenofovir Drug-Level Feedback Counseling Improves Long-Term Pre-exposure Prophylaxis Adherence for US Men Who Have Sex With Men in Pilot RCT

**DOI:** 10.1093/cid/ciag080

**Published:** 2026-02-10

**Authors:** Matthew A Spinelli, Leah Davis Ewart, Emily J Ross, Megan J Heise, Renata Buccheri, Carlos Moreira, Shivani Mahuvakar, David V Glidden, K Rivet Amico, Emily Arnold, Warren Rodrigues, Hideaki Okochi, Jennifer Manuel, Margaret Handley, Susan P Buchbinder, Adam Carrico, Monica Gandhi

**Affiliations:** Department of Medicine, University of California, SanFrancisco, California, USA; Department of Health Promotion and Disease Prevention, Florida International University, Miami, Florida, USA; Department of Health Promotion and Disease Prevention, Florida International University, Miami, Florida, USA; Department of Medicine, University of California, SanFrancisco, California, USA; Department of Medicine, University of California, SanFrancisco, California, USA; Department of Medicine, University of California, SanFrancisco, California, USA; Department of Medicine, University of California, SanFrancisco, California, USA; Department of Medicine, University of California, SanFrancisco, California, USA; Department of Health Behavior and Health Equity, University of Michigan, Ann Arbor, Michigan, USA; Department of Medicine, University of California, SanFrancisco, California, USA; Abbott Rapid Diagnostics, Pomona, California, USA; Department of Medicine, University of California, SanFrancisco, California, USA; Department of Medicine, University of California, SanFrancisco, California, USA; Department of Medicine, University of California, SanFrancisco, California, USA; Department of Medicine, University of California, SanFrancisco, California, USA; Bridge HIV, SanFrancisco Department of Public Health, San Francisco, California, USA; Department of Health Promotion and Disease Prevention, Florida International University, Miami, Florida, USA; Department of Medicine, University of California, SanFrancisco, California, USA

**Keywords:** PrEP, point-of-care, urine tenofovir, motivational interviewing, adherence intervention

## Abstract

A remote, motivational interviewing–informed intervention using self-administered point-of-care urine tenofovir testing improved long-term pre-exposure prophylaxis (PrEP) adherence among young US men-who-have-sex-with-men in a pilot randomized trial. Participants increased hair tenofovir levels by 1–2 tablets weekly, with high feasibility and acceptability, supporting further implementation.

Oral pre-exposure prophylaxis (PrEP) is highly effective, but its effectiveness is closely tied to adherence [1]. US young-men-who-have-sex-with-men (YMSM) experience high HIV-1 incidence and face persistent PrEP adherence challenges [2], including due to stigma, psychosocial stressors, or simply forgetting [3]. Furthermore, a drop-off in adherence may herald future PrEP discontinuation and signal a critical period for intervention in individuals who remain at risk [3]. Despite the development of long-acting injectable PrEP agents, oral PrEP accounts for 97–98% of PrEP use in the United States [4] and may be particularly important for younger populations who have difficulties with in-person healthcare visits due to busy work and school schedules.

Traditional adherence assessment relies on self-report, which is subject to social desirability and recall bias [5]. Objective, scalable adherence monitoring tools could support PrEP delivery and help to triage support to those who most need assistance. The urine-based point-of-care (POC) tenofovir (TFV) assay provides real-time results indicating recent dosing within the past 4–7 days for emtricitabine/tenofovir disoproxil fumarate (FTC/TDF) and within the last 1–2 days for FTC/tenofovir alafenamide (TAF [6, 7]). Results are available in 3 min, at low cost and with minimal training. A prior trial among PrEP-using women in Kenya found that integrating urine POC testing with motivational interviewing (MI)–informed drug-level feedback improved long-term adherence over 12 months [8].

However, evidence for POC adherence interventions among US YMSM is limited, and it is unknown whether remotely delivered, telehealth-based versions of drug-level feedback interventions are feasible or effective. We therefore conducted a pilot randomized controlled trial (RCT) of a remote, MI-informed adherence counseling intervention incorporating self-administered urine POC TFV testing among YMSM taking daily oral PrEP.

## METHODS

Between 11/2023 and 6/2024, we recruited participants via targeted advertisements on a geospatial social mobile application across the United States. Eligible participants were (1) male sex at birth <30 years old who have sex with men, (2) HIV-negative, (3) prescribed daily oral PrEP (FTC/TDF or FTC/TAF), and (4) willing to self-collect samples (urine/hair). This trial was registered on clinicaltrials.gov (NCT05353283; UCSF IRB#22-36616).

Participants provided electronic informed consent, completed baseline questionnaires, and were randomized 2:1 to intervention versus standard-of-care (SOC), with reimbursement provided for sample collection. Intervention participants received 2 MI-informed adherence counseling sessions with psychology post-doctoral students over a 3-month period over Zoom. At the beginning of each session, participants self-administered a mailed urine POC TFV test. Counselors provided feedback integrating test results and used MI strategies to explore barriers and support action planning during 2–20 min sessions 2–4 weeks apart. Participants could elect to discuss specific adherence barriers, including mental health, substance use, or partner influence. Standard-of-care participants continued routine PrEP care without urine POC testing or counseling. Fidelity to the intervention was monitored through video recording and review of all sessions (J. M.) using the Motivational Interviewing Treatment Integrity (MITI) tool [9]. Feasibility was assessed as percent urine tests performed successfully. Following each intervention session, participants completed brief questionnaires assessing perceived helpfulness of drug-level feedback.

### Adherence Measures

TFV hair drug levels served as the adherence outcome for this study, a long-term metric of adherence (lower quantification limit = 0.002 ng/mg [9, 10]). All participants self-collected hair samples at baseline and 3 months, mailing the samples to the UCSF Hair Analytical Laboratory. Tenofovir concentrations were quantified via liquid chromatography/tandem mass spectrometry (LC-MS/MS) using validated methods approved by the NIH's Clinical Pharmacology and Quality Assurance Program. Hair levels provide a validated measure of cumulative adherence over 4–6 weeks [8].

Point-of-care urine test positivity indicates TFV ingestion at a cutoff of 1500 ng/mL of TFV with results available in 3 min [6, 7].

### Statistical Analysis

The primary outcome was change in continuous TFV levels in hair between baseline and 3 months using mixed-effects linear regression to compare changes between groups, accounting for repeated measures and adjusting for baseline adherence. Secondary outcomes were descriptive and included urine test positivity and acceptability ratings. Exploratory analyses evaluated effects among participants with baseline suboptimal adherence (<4 doses/week [<0.023 ng/mg]). Analyses were performed in Stata 18.

## RESULTS

Of 63 participants randomized, 3 were excluded (2 inadequate hair samples; 1 lost to follow-up), yielding an analytic sample of 60 (40 intervention; 20 SOC) recruited across the United States. The median age was 27 years (inter-quartile range: 25–29); 35% were Hispanic, 25% were Black. Nearly half (47%) used daily FTC/TAF, while 53% used daily FTC/TDF (Supplementary Figures 1 and 2/Supplementary Table 1**)**. All participants reported ≥1 condomless anal sex partner. Moderate or greater substance use (WHO ASSIST score >3) was reported by 20%.

### Intervention Delivery and Acceptability

All 40 intervention participants successfully self-administered urine tests before both sessions and completed counseling. The average session length was 22.1 min (SD = 9.5). Counselors achieved “Good” or higher technical and relational intervention fidelity scores 87% (mean = 4.1; SD = 0.36) and 100% (mean = 4.6; SD = 0.36) of sessions respectively, with 78% of questions considered open versus closed questions. Reported acceptability was high: 93% found the intervention “somewhat/very” acceptable; 7% were neutral. No adverse events related to sample collection or counseling were reported.

### Adherence Outcomes

**Figure 1.**
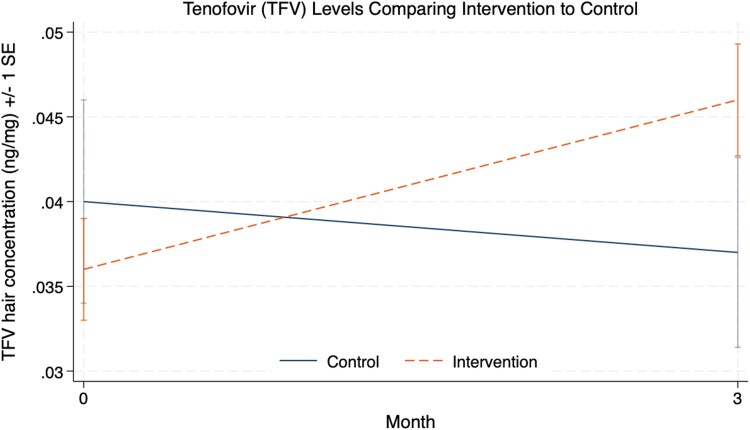
Hair tenofovir concentrations before and after the point-of-care urine tenofovir feedback counseling intervention. Hair tenofovir concentrations (ng/mg) are plotted at baseline and month 3 following the intervention. Control participants are plotted in solid blue while intervention participants are plotted in dotted orange. Error bars represent ±1 standard error (SE).

Over 3 months, intervention participants demonstrated greater increases in hair TFV levels than participants in the SOC arm (β = .01 ng/mg; 95% CI: .00–.02; *P* = .03; Figure 1). This increment corresponds to approximately consumption of 1–2 additional tablets per week based on established adherence thresholds. There was no difference in outcomes within the substrata of users of TAF versus TDF-based PrEP (interaction *P* = .52).

Among participants with baseline suboptimal adherence (<4 doses/week), 6/10 (60%) in the intervention arm improved above this threshold, compared with 0/5 in the SOC arm (*P* = .04).

Urine test positivity in the intervention group increased from 37/40 (93%) at baseline to 40/40 (100%; *P* = .13) at follow-up. Notably, there was only one participant with hair TFV near limit of detection who had a positive urine test. However, his follow-up hair sample indicated improvement to ∼3 doses/week (0.020 ng/mg).

## DISCUSSION

In this pilot RCT, self-administered urine POC TFV testing followed by a remote adherence counseling intervention incorporating POC adherence self-testing improved biologically verified PrEP adherence among YMSM in the United States. This study supports the feasibility, acceptability, and preliminary effectiveness of integrating objective, real-time drug-level feedback into telehealth-delivered PrEP support for the first time.

The intervention resulted in participants increasing ingestion of PrEP by 1–2 additional PrEP doses per week, a clinically significant increase. Prior pharmacologic modeling indicates that 4 times weekly doses of TFV provide ∼99% protection against HIV acquisition in MSM [1]. Our exploratory analysis showed that 60% of participants with suboptimal adherence improved above this threshold with the intervention, versus none in the control group.

High acceptability and improved long-term adherence among participants support the potential for scalability of the intervention, particularly as PrEP care increasingly leverages telehealth [11]. Integration of such interventions could help address disparities in adherence and persistence among YMSM or other populations disproportionately impacted by HIV, particularly those who live in rural areas or with limited healthcare access.

Our findings extend prior work from Kenya, where clinic-based feedback on urine TFV results improved long-term PrEP adherence (assessed via hair levels of TFV) among young women [8], and viral suppression among people with HIV at public health clinics in Namibia [12]. By demonstrating feasibility of telehealth-delivered urine TFV POC drug-level feedback among YMSM, this study suggests that drug-level feedback interventions may be adaptable across populations and delivery models. Furthermore, this is the first study to perform drug-level feedback among users of FTC/TAF-based PrEP, with no difference in outcomes compared to FTC/TDF.

Although it is possible that white coat adherence, where participants increase adherence transiently prior to clinical visits, occurred, the only participant with a pattern consistent with white coat adherence subsequently increased to 3 tablets weekly on follow-up. It is also possible that the desire to demonstrate improved adherence at clinical visits may lead to longer-term impacts on adherence behavior which would be beneficial.

While long-acting injectable PrEP medications have outstanding promise for improving PrEP uptake and persistence, studies to date have shown that oral PrEP remains the predominant form prescribed due to cost and structural factors limiting access [4]. Long-acting lenacapavir, while limiting the number of injections to 2 annually, is unfortunately delivered at the same yearly cost as cabotegravir. Scalable strategies which enhance the efficacy of oral PrEP are likely to continue to be needed in a time of declining national prevention and healthcare funding. Moreover, remotely delivered interventions and low-cost scalable adherence tools could translate well to global health settings.

Limitations of this study include the small sample size, short follow-up, inability to confirm HIV status at baseline or downstream HIV incidence, and exclusion of on-demand PrEP. Additionally, generalizability may be limited to YMSM engaged via app-based recruitment and willing to self-collect samples. Larger, longer-term RCTs should be completed next to establish efficacy and implementation strategies, such as comparing in-clinic versus video delivery with or without counseling.

If confirmed in larger trials, POC urine TFV feedback could be integrated into national and international PrEP programs as a low-cost, scalable adherence support tool. Its compatibility with remote counseling is particularly relevant in the current telehealth landscape and with mobile or rural populations. Broader implementation could enhance PrEP effectiveness at the population level.

## Supplementary Material

ciag080_Supplementary_Data

## Supplementary Data

Supplementary materials are available at *Clinical Infectious Diseases* online. Consisting of data provided by the authors to benefit the reader, the posted materials are not copyedited and are the sole responsibility of the authors, so questions or comments should be addressed to the corresponding author.
