# Supplementary material for: Point-of-Care Urine Tenofovir Drug-Level Feedback Counseling Improves Long-Term Pre-exposure Prophylaxis Adherence for US Men Who Have Sex With Men in Pilot RCT
Source: Clin Infect Dis. 2026 Feb 10;82(5):e992–5. doi: 10.1093/cid/ciag080 (PMC13189661; doi:10.1093/cid/ciag080)
Supplement: ciag080_Supplementary_Data [file ciag080_supplementary_data.docx]

**Supplemental Methods:**

**Sample Size Calculation:** Using an estimate of PrEP2-BAY being acceptable for 90% of the 40 individuals in the intervention arm, we would have 82% power to detect acceptability of 75% or more with a one-sided alpha of 0.015

**Motivational Interviewing Fidelity:** Motivational Interviewing fidelity scoring was completed using the Motivational Interviewing Integrity Code (Moyers TB, Rowell LN, Manuel JK, Ernst D, Houck JM. The Motivational Interviewing Treatment Integrity Code (MITI 4): Rationale, Preliminary Reliability and Validity. J Subst Abuse Treat. 2016 Jun;65:36-42). All video recordings of visits were reviewed by an experienced evaluator (Dr. Jen Manuel), who is one of the developers of this scale.

**Hair Tenofovir Dosing Cut-offs:** A cut-off of 0.023 ng/mg was used to represent 4-times-weekly dosing based on the Tenofovir Strand Study (Liu AY et al. PLoS One. 2014 Jan 8;9(1):e83736), which was the median hair tenofovir concentration of individuals receiving tenofovir disoproxil fumarate/emtricitabine with this pattern using directly observed therapy. Despite much lower plasma concentrations with tenofovir alafenamide/emtricitabine, hair concentrations are similar when compared to tenofovir disoproxil fumarate dosing (Nerguizian D. et al. British Journal of Clinical Pharmacology. 2024;90(S1):15-16; Okochi H et al. Drug Test Anal. 2021 Jul;13(7):1354-1370).

**Supplemental Table:**

|  |  | **Control** | | | **Intervention** | | **Overall** | | | ***p*-value*** |
| --- | --- | --- | --- | --- | --- | --- | --- | --- | --- | --- |
|  |  | N = 20 | % | | N = 40 | % | N = 60 | | % |  |
| Age, median [IQR] | | 27 [25, 28] | | | 27.5 [25, 29] | | 27 [25, 29] | | | .40 |
| Ethnicity | |  | |  |  |  |  |  | |  |
|  | Non-Hispanic | 13 | | 65% | 26 | 65% | 39 | 65% | | 1.00 |
|  | Hispanic or Latino | 7 | | 35% | 14 | 35% | 21 | 35% | |  |
| Race | |  | |  |  |  |  |  | |  |
|  | White | 10 | | 50% | 21 | 53% | 31 | 52% | | .66 |
|  | Multiracial or other | 2 | | 10% | 2 | 5% | 4 | 7% | |  |
|  | Asian | 2 | | 10% | 8 | 20% | 10 | 17% | |  |
|  | Black | 6 | | 30% | 9 | 23% | 15 | 25% | |  |
| Gender/sexual orientation | |  | |  |  |  |  |  | |  |
|  | Man who has sex with men | 18 | | 90% | 37 | 93% | 55 | 92% | | .74 |
|  | Genderqueer (male sex at birth) who has sex with men | 2 | | 10% | 3 | 8% | 5 | 8% | |  |
| Region | |  | |  |  |  |  |  | |  |
|  | West | 11 | | 55% | 17 | 43% | 28 | 47% | | .29 |
|  | South | 2 | | 10% | 12 | 30% | 14 | 23% | |  |
|  | Northeast | 3 | | 15% | 7 | 18% | 10 | 17% | |  |
|  | Midwest | 4 | | 20% | 4 | 10% | 8 | 13% | |  |

*Kruskal-Wallis tests were performed for continuous measures (e.g., age), and Chi-square tests were performed for categorical measures (e.g., ethnicity).

**Supplemental Figure 1:**

**Legend:** A Consort Diagram shows the proportion assessed for eligibility for the PrEP2-BAY Study, those who were excluded, those who were randomized, those who received the intervention, and those who were analyzed.

**
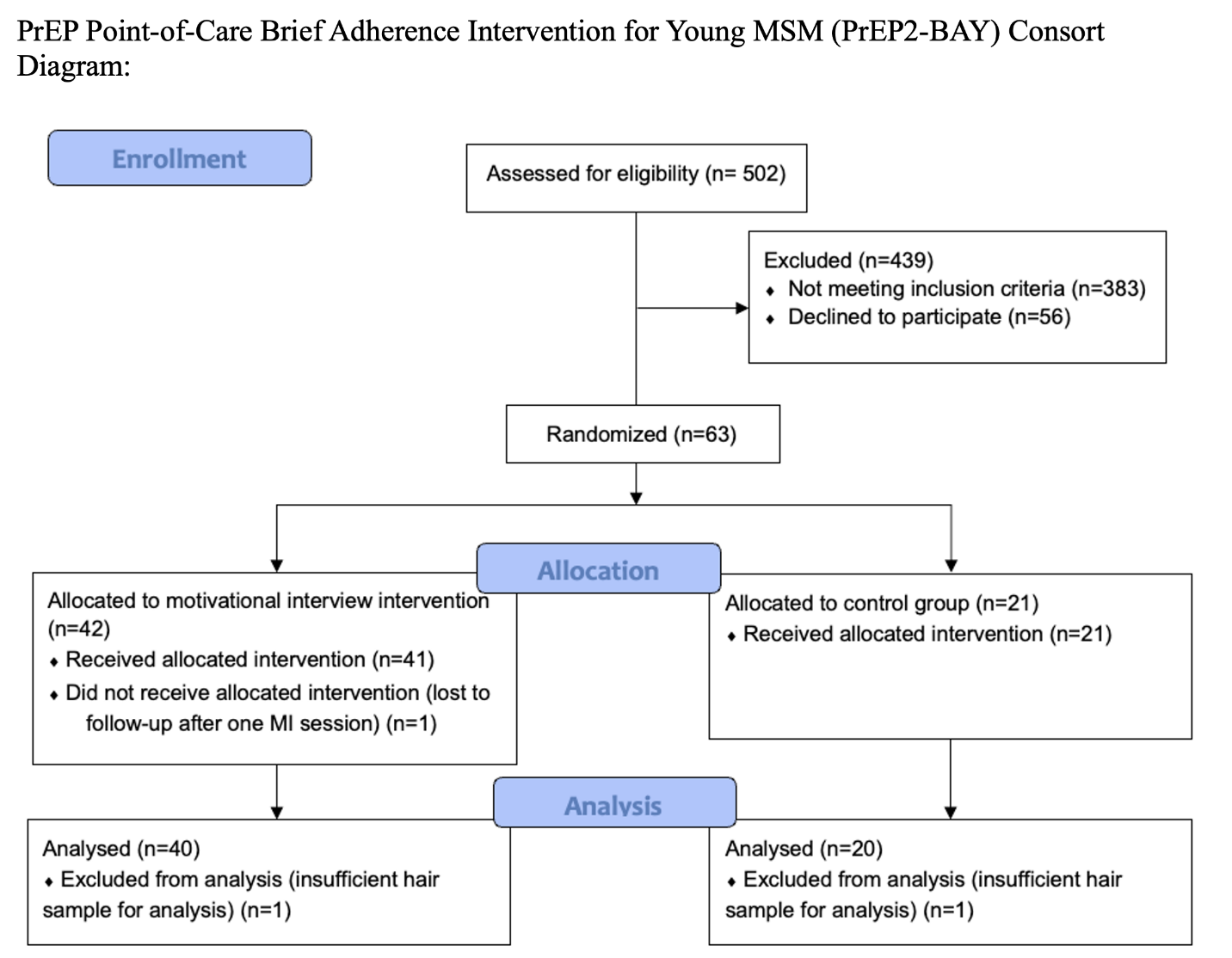
**

**Supplemental Figure 2: Map of Participant Locations by Intervention or Control Arm
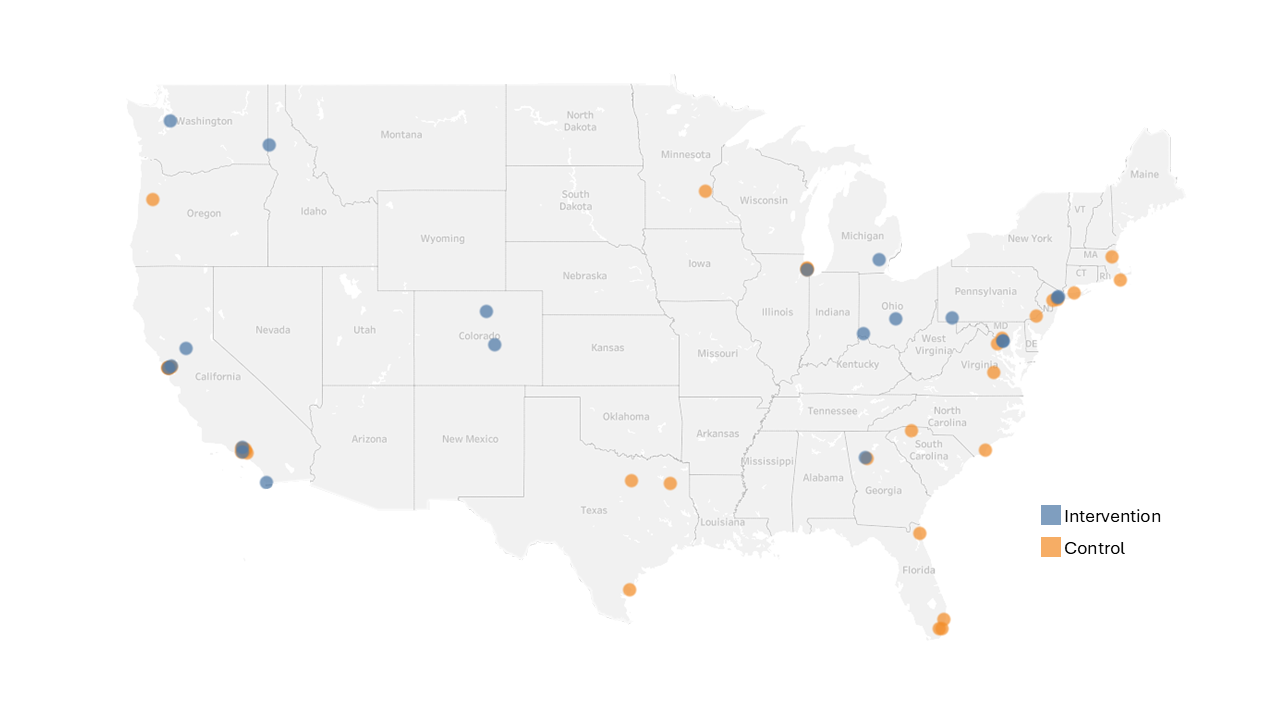
**
